# Liquid water in the Martian mid-crust

**DOI:** 10.1073/pnas.2409983121

**Published:** 2024-08-12

**Authors:** Vashan Wright, Matthias Morzfeld, Michael Manga

**Affiliations:** ^a^Scripps Institution of Oceanography, University of California San Diego, La Jolla, CA 92093; ^b^Department of Earth and Planetary Science, University of California Berkeley, Berkeley, CA 94720

**Keywords:** Mars, water, planetary geophysics, InSight

## Abstract

Large volumes of liquid water transiently existed on the surface of Mars more than 3 billion years ago. Much of this water is hypothesized to have been sequestered in the subsurface or lost to space. We use rock physics models and Bayesian inversion to identify combinations of lithology, liquid water saturation, porosity, and pore shape consistent with the constrained mid-crust (∼11.5 to 20 km depths) seismic velocities and gravity near the InSight lander. A mid-crust composed of fractured igneous rocks saturated with liquid water best explains the existing data. Our results have implications for understanding Mars’ water cycle, determining the fates of past surface water, searching for past or extant life, and assessing in situ resource utilization for future missions.

Liquid water existed at least episodically on Mars in rivers (1), lakes (1), oceans (2), and aquifers (3) during the Noachian and Hesperian, more than 3 billion years ago. Mars lost its ability to host persistent bodies of liquid water on its surface after the planet lost most of its atmosphere during this time period (4). The ancient surface water may have been incorporated in minerals (5), buried as ice, sequestered as liquid in deep aquifers, or lost to space (4).

Geophysical measurements have the potential to identify water in the deep subsurface. For example, seismic velocities derived from ground motion measured by the InSight (interior exploration using seismic investigations, geodesy, and heat transport) mission and interpreted with rock physics models have been used to constrain water distribution to depths of 20 km beneath the InSight lander, Elysium Planitia. The shear *V*_*s*_ and compression *V*_*p*_ wave velocities within the upper 300 m beneath InSight are consistent with a dry crust composed of minimally cemented (<2% of the pores) sediments (6). *V*_*s*_ in the upper 8 km beneath InSight is lower than expected for an ice-saturated cryosphere (7), though *V*_*s*_ may be higher elsewhere (8, 9). Kilburn et al. (7) argue that the crust between 8 and 20 km beneath InSight is a) mafic and highly porous or b) felsic and less porous, but with *V*_*s*_ alone, could not determine whether the fractures contain liquid water.

We assess whether *V*_*s*_ (10–13), *V*_*p*_ (12), and bulk density *ρ*_*b*_ (14) data (Table 1) are consistent with liquid water-saturated pores in the mid-crust (11.5 ± 3.1 to 20 ± 5km) within 50 km of the InSight lander. The mid-crust is one of four robust seismically detectable kilometer-scale layers beneath InSight (10–13) and may be global (8). *V*_*p*_ and layer thickness have been challenging to obtain for other locations on Mars (see ref. 9 and references therein). Temperatures on present-day Mars become warm enough for stable liquid water near the top of mid-crust (15), and pores are expected to have closed at the bottom of the layer (16). We use Bayesian inversion and a Markov chain Monte Carlo (MCMC) algorithm (17) to identify combinations of six lithologic parameters (pore shape aspect ratio *α*, porosity *ϕ*, liquid water saturation *γ*_*w*_, mineral bulk modulus *κ*_*m*_, mineral shear modulus *μ*_*m*_, mineral density *ρ*_*m*_, Table 2) that best reproduce the three observed data points *V*_*p*_, *V*_*s*_, and *ρ*_*b*_ (Table 1). Calculations combine the seismic velocity equations, the Berryman self-consistent rock physics model (18), and the Gassmann–Biot equations (19) (*Materials and Methods*). A mid-crust composed of igneous rock with thin fractures filled with liquid water can best explain the geophysical data.

**Table 1.** Geophysical data for the mid-crust beneath the InSight lander

Source	*V*_*p*_ (km/s)	*V*_*s*_ (km/s)	*ρ*_*b*_ (kg/m^3^)
Knapmeyer-Endrun et al. (10)	—	2.3±0.3	—
Duran et al. (11)	—	2.5 to 3.3	—
Carrasco et al. (12)	3.75 to 4.55	2.0 to 2.5	—
Joshi et al. (13)	—	2.3 to 2.6	—
Derived from refs. 14, 26, and 27	—	—	2,589±157

See *Materials and Methods* for *ρ*_*b*_ calculations, which assume crustal mineralogies ranging between 100% plagioclase and 100% basalt.

**Table 2.** Model parameters (7) explored in the inversion

Parameters	Ranges
Pore shape aspect ratio (*α*)	0.03 to 0.99
Porosity (*ϕ*)	0.05 to 0.50
Water saturation (*γ*_*w*_) (%)	0 to 100
Mineral bulk modulus (*κ*_*m*_) (GPa)	76.5 to 80
Mineral shear modulus (*μ*_*m*_) (GPa)	25.6 to 40
Mineral density (*ρ*_*m*_) (kg/m^3^)	2,689 to 2,900

## Results and Discussion

**Fig. 1.**
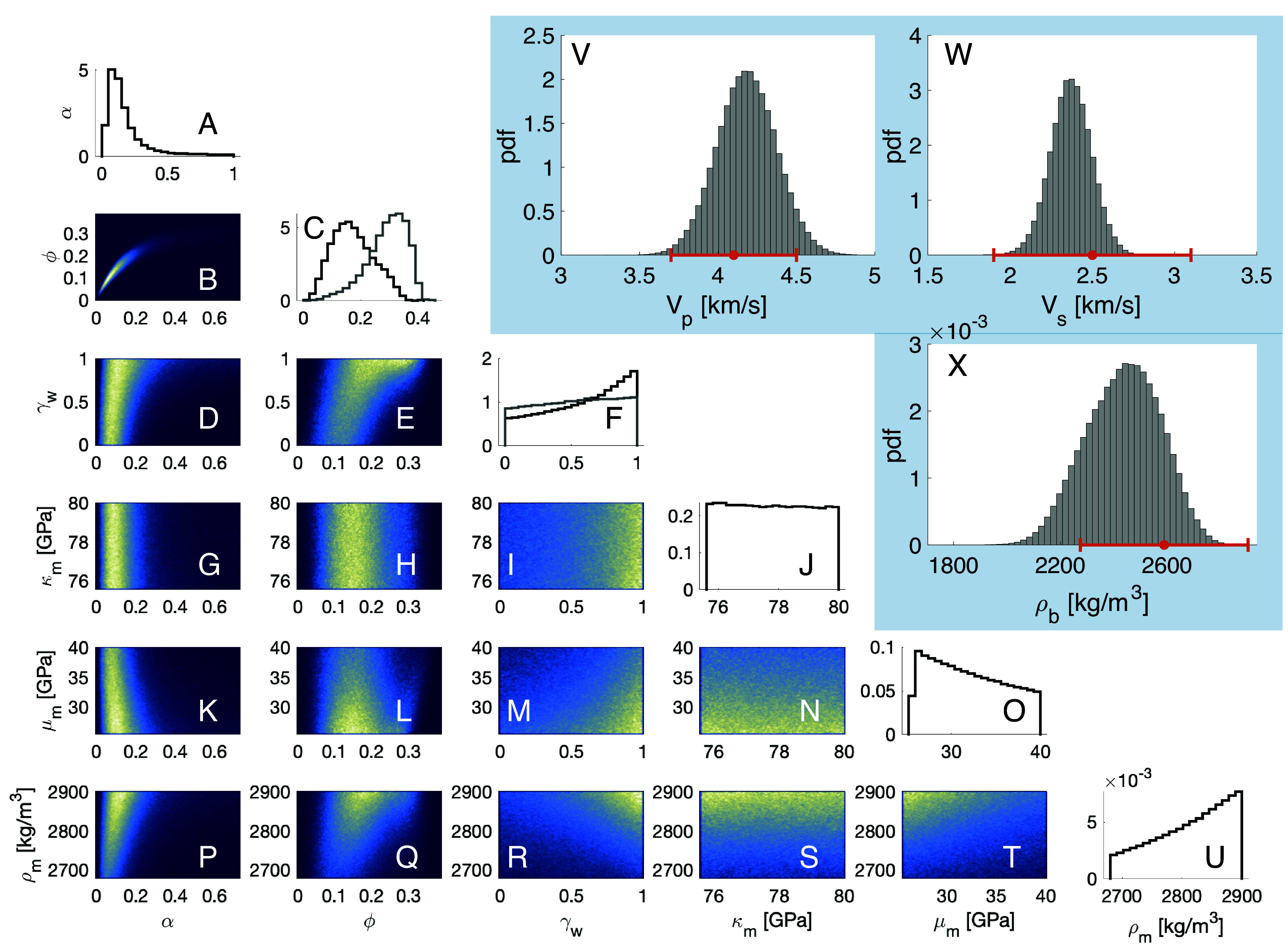
Summary of inversion results. Panels (*A*–*U*): Histograms of marginal posterior distributions of model parameters, computed from $5\times 10^{5}$ iterations of the MCMC (17). The area under each histogram is equal to one. In the 2D histograms, cold colors (blues) indicate low posterior probability, and warm colors (yellows and whites) indicate regions of high posterior probability. In the 1D histograms, black stair plots show results for our default parameters bounds (Table 2). The light gray stair plots in panels (*C*) and (*F*) illustrate results obtained with widened bounds on mineralogical parameters (*Results and Discussion*). Water content is nearly uniformly distributed (*F*) under these assumptions, but the porosity takes on unreasonably large values (0.29±0.07). Panels (*A*) and (*C*) show that *α* and *ϕ* are tightly constrained by the data. Panel (*B*) reveals a nonlinear relationship between *ϕ* and *α*. Panel (*F*) indicates that a high water saturation is likely in view of the data. Panel (*J*) shows that *κ*_*m*_ is not constrained by the data. Panels (*V*–*X*): Data fits. Histograms show model responses (*V*_*p*_, *V*_*s*_, and *ρ*_*b*_) for each of the parameters in panels (*A*–*U*), normalized so that the area under the graph is one. The orange error bars (horizontal) illustrate the mean of the data (filled dot) and expected errors (two SD).

Fig. 1 summarizes inversion results when the MCMC algorithm samples a range of mineral moduli and densities spanning from mafic (14, 20) to more evolved igneous rocks (14, 21) represented by a range between 100% basalt and 100% plagioclase. Several combinations of parameters produce good fits to the observed *V*_*p*_, *V*_*s*_, and *ρ*_*b*_ data within assumed errors (Fig. 1*V*–*X*). *α*, *ϕ*, *μ*_*m*_, and *γ*_*w*_ are well resolved. A fully liquid water-saturated crust $\gamma_{w}=100\%$ is most probable (Fig. 1*F*); *ϕ* is estimated as 0.17±0.07 (Fig. 1*C*) and *α* as 0.19±0.18 (Fig. 1*A*), implying thin fractures. The inversion recovers a nonlinear relationship between *α* and *ϕ* (Fig. 1*B*). *κ*_*m*_ is not well-constrained by the data (Fig. 1*K*).

We explored the robustness of the result above by expanding the mineral parameter bounds the MCMC explores: $\rho_{m}=2,680-4,250$ kg/m^3^, $\kappa_{m}=75.6-107.6$ GPa, and $\mu_{m}=25.6-76.8$ GPa. $\gamma_{w}=100\%$ remains most probable until the MCMC explores $\rho_{m}>4,000$ kg/m^3^. $\gamma_{w}=0-100\%$ becomes nearly equally probably beyond this (Fig. 1*F*), also resulting in ϕ=0.29±0.07 (Fig. 1*C*) and $\rho_{m}=3,702\pm 363$ kg/m^3^. This latter solution is inconsistent with independent observations: i) ϕ=0.29±0.07 is larger than the mean *ϕ* of the crust (0.1 to 0.23) (22, 23) and dense (>3,100 kg/m^3^) Martian meteorites (∼0.1, with values typically <0.23) (24); ii) *ϕ* at the surface is 0.3 to 0.5 (25, 26) and should substantially decrease at mid-crustal depths, with pores closing at 20 km as discussed in refs. 7, 14, 16, 23, and 26.

A mid-crust containing liquid water has implications for the Martian water budget and hydrological cycle. Assuming the InSight location as representative, motivated by similar $V_{p}/V_{s}$ (1.81 to 1.98) and seismically derived *ϕ* (0.1 to 0.17) (8) beneath InSight and areas up to 4,500 km away from the lander, 10 km of crust with porosity of 0.1 to 0.2 translates to 1 to 2 km of water—more than the water volumes proposed to have filled hypothesized ancient Martian oceans (2). Thus, Mars’ crust need not have lost most of its water via atmospheric escape. Liquid water in the pores of the mid-crust also requires high enough permeability and warm enough temperatures in the shallow crust to permit exchange between the surface and greater depths. While available data are best explained by a water-saturated mid-crust, our results highlight the value of geophysical measurements and better constraints on the mineralogy and composition of Mars’ crust.

## Materials and Methods

### Constraining the Mid-Crustal Bulk Density.

The bulk density of the mid-crust has not been directly constrained by the gravity, seismic velocity, and mineralogical data used to derive the average bulk density and thickness of the crust beneath InSight (14). We can, however, infer the bulk density of the mid-crust using three constraints. First, the average bulk density within the upper 1.2 km is 1,600±360 kg/m^3^ and 2,300±130 kg/m^3^ between 1.2 and 11.5 km. These numbers are based on the estimated average bulk densities within the upper few hundred meters below the surface (26) and ∼5 km below the surface (27) of the adjacent Gale Crater on Mars. Second, the bulk density of the crust increases with depth (22). Third, the bulk density of the layer beneath 20 km ± 5 km is the same as its mineral density due to pore-closure (16). An average bulk density of the mid-crust can be obtained by solving a constrained problem to reproduce the average bulk density of the crust, 2,580±209 kg/m^3^ (14).

### Rock Physics Models.

Seismic velocities *V*_*p*_ and *V*_*s*_ depend on bulk density *ρ*_*b*_ and effective shear *μ*_*e*_ and bulk *κ*_*e*_ moduli:

[1] $$\begin{array}{c} V_{p}={\left(\left(\kappa_{e}+\left(4/3\right)\mu_{e}\right)/\rho_{b}\right)}^{1/2},V_{s}={\left(\mu_{e}/\rho_{b}\right)}^{1/2} \end{array}$$

Berryman’s rock physics model (18) provides dry-frame shear *μ*_*d*_ and bulk *κ*_*d*_ moduli of fractured rocks [see ref. 7 for a list of Berryman’s equations (18)]. The model uses a self-consistent approach and long-wavelength scattering theory that allows inclusions to overlap (18). Model inputs are *ϕ*, *κ*_*m*_, *μ*_*m*_, *ρ*_*m*_, and *α*. *μ*_*e*_ = *μ*_*d*_ (19).

We use Gassmann–Biot fluid substitution theory (19) to estimate *κ*_*e*_ from *κ*_*d*_, *ϕ*, *κ*_*m*_, and the bulk moduli of the fluid in a dry ($\kappa_{f1}$ = 0 kPa for gas) versus partially to fully liquid-saturated ($\kappa_{f2}$) rock,

[2] $$\begin{array}{c} \frac{\kappa_{e}}{\kappa_{m}-\kappa_{e}}-\frac{\kappa_{f2}}{\phi (\kappa_{m}-\kappa_{f2})}=\frac{\kappa_{d}}{\kappa_{m}-\kappa_{d}}+\frac{\kappa_{f1}}{\phi (\kappa_{m}-\kappa_{f1})}. \end{array}$$

With constraints on *μ*_*e*_ and *κ*_*e*_ from Berryman and Gassmann–Biot equations (18, 19), we then estimate *V*_*s*_ and *V*_*p*_ via Eq. **1**.

### Bayesian Inversion.

We perform a Bayesian inversion, which requires that we specify a prior $p_{0}\left(x\right)$ and a likelihood $p_{l}\left(y|x\right)$, where *x* are the six unknown parameters that we invert for ($\alpha ,\phi ,\gamma_{w},\kappa_{m},\mu_{m}$, and *ρ*_*m*_, which control *κ*_*e*_, *μ*_*e*_, and *ρ*_*b*_) and

[3] $$\begin{array}{c} y=(4.1\;\text{km/s},2.5\;\text{km/s},2,589\;{\text{kg/m}}^{3}), \end{array}$$

are the three data (*V*_*p*_, *V*_*s*_, and *ρ*_*b*_) we seek to explain. The prior is a uniform distribution over the parameter bounds in Table 2, combined with the constraint that $V_{p}>V_{s}$. The likelihood follows from assuming Gaussian errors in the data

[4] $$\begin{array}{c} p\left(y|x\right)\propto \;\exp\left(-{0.5\|W\left(y-m\left(x\right)\right)\|}_{2}^{2}\right), \end{array}$$

where m(x) is the rock physics model (i.e., the forward model) and where *W* is a diagonal matrix whose diagonal elements are the reciprocals of the standard deviations of the data ($\sigma_{V_{p}}=0.2$ km/s, $\sigma_{V_{s}}=0.3$ km/s, $\sigma_{\rho_{b}}=157\;{\text{kg/m}}^{3}$, derived from Table 1 to render all reported data points as likely). Jointly, the prior and likelihood define a Bayesian posterior distribution, $p\left(x|y\right)\propto p_{0}\left(x\right)p_{l}\left(y|x\right)$, which we sample via an affine invariant MCMC ensemble sampler (17). Sensitivity analyses confirm that water saturation does not significantly influence *V*_*s*_ (19) and most strongly influences the *V*_*p*_, followed by *ρ*_*b*_ (18).

## Data Availability

Published data were analyzed in this study (10–14). Matlab scripts to reproduce this work or consider new data and constraints are at https://github.com/mattimorzfeld/WMM24.
